# Colorectal Cancer Screening: Tests, Strategies, and Perspectives

**DOI:** 10.3389/fpubh.2014.00210

**Published:** 2014-10-27

**Authors:** Fabrizio Stracci, Manuel Zorzi, Grazia Grazzini

**Affiliations:** ^1^Public Health Section, Department of Experimental Medicine, University of Perugia, Perugia, Italy; ^2^Regional Cancer Registry of Umbria, Perugia, Italy; ^3^Registro Tumori del Veneto, Padova, Italy; ^4^Department of Screening, ISPO Cancer Prevention and Research Institute, Florence, Italy

**Keywords:** colorectal cancer, advanced adenoma, screening, flexible sigmoidoscopy, total colonoscopy, fecal tests, CT colonography, capsule endoscopy

## Abstract

Screening has a central role in colorectal cancer (CRC) control. Different screening tests are effective in reducing CRC-specific mortality. Influence on cancer incidence depends on test sensitivity for pre-malignant lesions, ranging from almost no influence for guaiac-based fecal occult blood testing (gFOBT) to an estimated reduction of 66–90% for colonoscopy. Screening tests detect lesions indirectly in the stool [gFOBT, fecal immunochemical testing (FIT), and fecal DNA] or directly by colonic inspection [flexible sigmoidoscopy, colonoscopy, CT colonography (CTC), and capsule endoscopy]. CRC screening is cost-effective compared to no screening but no screening strategy is clearly better than the others. Stool tests are the most widely used in worldwide screening interventions. FIT will soon replace gFOBT. The use of colonoscopy as a screening test is increasing and this strategy has superseded all alternatives in the US and Germany. Despite its undisputed importance, CRC screening is under-used and participation rarely reaches 70% of target population. Strategies to increase participation include ensuring recommendation by physicians, introducing organized screening and developing new, more acceptable tests. Available evidence for DNA fecal testing, CTC, and capsule endoscopy is reviewed.

## Role of Secondary Prevention in Colorectal Cancer Control

Screening has a central role in colorectal cancer (CRC) control. Even if incidence studies show that the majority of large bowel cancers are sporadic, few protective and risk factors for the disease have been consistently identified so far (e.g., alcohol, red and processed meat intake, obesity, smoking, physical activity, and aspirin use) (1). Modifiable factors are associated with slightly elevated risks of developing CRC, in most cases between 1.2 and 2.0. Some established risk factors are widely diffused in the general population so that they are responsible for a substantial share of disease burden and, thus, are potential targets for preventive interventions (2). However, changing dietary habits and physical activity levels is difficult. Typically, the Western lifestyle is associated with an unfavorable pattern of risk factors and with high CRC incidence rates (3, 4) and these, together with mortality trends, are also reported in developing countries (5, 6). CRC prognosis has slowly been improving (7). Progress has been made in the treatment of the disease, particularly for rectal cancer (8). However, both advanced age and stage at diagnosis limit the opportunity for curative treatment in many cases (9). Diagnosis at earlier stages in disease development has led to a dramatic change in prognosis and to more conservative treatment (10). Thus, screening is presently the key intervention for CRC control.

## Disease is Suitable for Screening

Colorectal cancer is an ideal target for screening interventions (11).

Colorectal cancer is an important health burden. With 746,000 cases in males (10.0% of total cancer cases) and 614,000 cases in women (9.2% of total), large bowel cancer is estimated to be the third (following lung and breast cancer) most frequent cancer worldwide in both sexes combined (12). In western countries, CRC is the most frequent cancer in both sexes (13). CRC is also a leading cause of cancer death, ranking fourth after lung, liver, and stomach cancer (12).

In the majority of cases, the disease develops over many years through the so-called adenoma–carcinoma sequence (14). Detection and removal of pre-malignant lesions can prevent progression to cancer and decrease incidence (15–18). CRC, as with many other cancers, is curable in most cases if detected at an early stage (10).

## Colorectal Cancer Screening Tests

Screening tests are available to detect pre-malignant lesions and cancer at early stages. Screening tests are classified in two groups: (i) indirect tests looking for the presence of markers of colorectal neoplasm in the stool; (ii) tests based on direct visualization of neoplasm in the large bowel. Currently, both stool-based [i.e., fecal occult blood test (FOBT) and fecal immunochemical test (FIT)] and endoscopy-based tests [i.e., flexible sigmoidoscopy (FS) and total colonoscopy (TC)] are almost exclusively used in ongoing opportunistic and organized CRC screening worldwide.

There is evidence from randomized controlled trials (RCT) and meta-analyses that guaiac-based [guaiac-based fecal occult blood testing (gFOBT)] and FS screening decrease CRC-specific mortality (15, 19). FIT shares the same indirect target for the presence of colorectal neoplasm with gFOBT; that is, the presence of blood in the stool. There is sufficient evidence that FIT outperforms gFOBT in terms of sensitivity (20, 21) and compliance with invitation (22). There is no available evidence from RCTs supporting the efficacy of colonoscopy as a CRC screening test and results from the ongoing RCTs will take decades to appear (23–25). However, TC is considered an effective screening test for CRC based on the following arguments: (i) evidence available for FS applies to TC as well since both tests are based on direct visualization of intestinal lumen; (ii) mortality reduction achieved with FOBT tests depends on colonoscopy as the confirmatory test; and (iii) available observational studies confirm that TC is highly effective in reducing CRC mortality and incidence (26). CRC screening is also cost-effective if compared to no screening (27, 28). Screening tests currently in use have recently been reviewed in detail (15, 21, 29).

## Guaiac-Based Fecal Occult Blood Test

Four large RCTs (30–33) showed that a screening program based on a gFOBT repeated every 1 or 2 years reduces CRC-specific mortality by 16% (up to 23% according to the per-protocol analysis) (34).

Some characteristics of gFOBT may limit compliance: they must be collected from three consecutive stools and they are sensitive to diet intake of hemoglobin (35). This led to the indication of dietetic restrictions at the price of loss in compliance; diet indications were subsequently dropped because of limited clinical significance (36–39). gFOBT is also sensitive to bleeding of the upper gastro intestinal (GI) tract (40). A further limitation of gFOBT is its low sensitivity for CRC (25–38%) and for advanced adenomas (AA) (16–31%) (20), the latter probably being the reason for the lack of incidence reduction among screened populations (34). Test sensitivity may be increased by rehydrating the test windows before development (41), but this significantly affects specificity (39, 42). Since the reading of the test is not automated, an inter-operator as well as a batch-to-batch variability has been reported (39). gFOBT is more stable than FIT if exposed to high temperature (43).

## Fecal Immunochemical Test

No RTC on the impact of FIT-based screening on CRC mortality has been carried out. A recent ecological study showed a 22% reduction in CRC-specific mortality in areas where FIT screening programs were active compared to controls (44). The impact of screening with FIT on incidence rates has also been reported (18). Further evidence of FIT test efficacy is indirect, based on a large number of trials comparing performance of different FIT tests with gFOBT (45–54). These studies are difficult to compare because they used different positivity cut-offs and different sample numbers (51–53, 55). Overall sensitivity of FIT is higher than gFOBT both for CRC (61–91%) and for AA (27–67%) and the test has a comparable specificity (ranging from 91 to 98% versus 98–99%) (40).

Fecal immunochemical tests are specific for human blood and insensitive to upper GI bleeding (21). There are qualitative FIT tests that produce a binary result (positive or negative), whose positivity cut-off may not be adjusted and whose reading is not automated. Their performance showed a large variability among manufacturers (56). Quantitative FIT produce a quantitative result of the fecal hemoglobin concentration, generally as nanograms of hemoglobin per milliliter buffer added and whose positivity cut-off may be adjusted.

The best number of samples and the positivity cut-off have not yet been defined and this uncertainty reflects on recommendations published in different countries (45, 50, 57–60). However, complicated stool handling may negatively affect screening compliance; according to two studies, 60–62% with 1-sample FIT versus 47–50% with three-samples gFBOT (50, 52). In addition, recent findings show that increasing the number of FIT samples does not affect the main performance indicators of FITs for CRC (61). It is thus reasonable to use a single sampling and to act on the cut-off to adjust the desired sensitivity (22, 62). Some trials have identified a protocol based on a single sample with a positivity cut-off of 75 ng/ml as a good trade-off between sensitivity and specificity (49, 63–65). Storage conditions (e.g., excessive temperature) may increase false-negative rates (64) and require technology solutions by the manufacturers. According to some recent studies, FIT is the most cost-effective tool for CRC screening (66–68) with a sensitivity (76%) and a specificity (95%) comparable to TC (24).

The European Guidelines recommend FIT for programs adopting a strategy based on fecal occult blood test (21).

## Flexible Sigmoidoscopy

Randomized controlled trials showed that screening with FS reduces CRC mortality by 22–31% and incidence by 18–23% through visual inspection of colic mucosa, biopsy taking and polyp removal in the distal tract of colon (69–71). A population-based trial showed similar results after a median follow up of 10.9 years (72).

The impact of screening with FS on incidence and mortality rates is limited to the distal colon, while RCTs showed no significant differences as regards the distal tract. A combined strategy using FS and gFOBT/FIT did not seem to solve the problem (72, 73) while it would increase endoscopic workload and reduce participation (72, 74, 75).

Overall, FS is a safe test: very low complication rates (mainly perforations and bleeding requiring transfusion) have been reported (76).

## Total Colonoscopy

Total colonoscopy allows direct visualization of the colonic mucosa, biopsy of lesions, and polyp removal over the whole colon. Sensitivity and specificity for CRC and AA are very high, even if a miss-rate of CRC of between 0.2 and 5.0% was reported (77–82). TC is the confirmatory test used in case of a positive test for all the above screening strategies (21, 42, 83).

Evidence of efficacy derives from observational studies, showing a relevant impact on incidence (reduced by 66–90% compared to the general population) and mortality (31–65%) (16, 84–87).

Total colonoscopy as a CRC screening test is not free from limits. A high inter-operator variability in the adenoma detection rate has been reported and this feature has been associated with the subsequent risk of CRC (88). Moreover, retrospective analyses questioned the capability of reducing incidence and mortality from proximal CRC in community settings (89, 90). Thus, proper training programs for endoscopists are necessary, as well as continuous quality assurance (91). Collateral effects of TC are rare but more frequent than with FS (20).

Many characteristics negatively affect the acceptability of TC as a first-line screening test: it is an invasive examination, which requires an even more invasive bowel cleansing and it is time-consuming, expensive, and painful. Even if in the US the uptake of TC is increasing (92), in the EU countries compliance has been very low (93).

## Geographic Distribution of CRC Screening

Diffusion of CRC screening shows a marked geographic variation (94). Screening is more frequently available in high incidence, high resources western countries. CRC screening practices in Europe have recently been reviewed by Altobelli et al. (13). Stool-based tests are more used than endoscopy. gFOBT is the recommended test in many countries like UK, France, and Finland. FIT is the test of choice in Spain, the Netherlands, and in most Italian programs. Screening is mainly national and programed in European countries and includes the 50–74 age group as the target population. Opportunistic screening is diffused in Austria and Germany, and, outside Europe, in Japan, Australia, and Canada (95). Germany, Poland, and the US adopt TC as CRC screening test, alone or as an alternative to other possible test choices. The US has a longstanding history of CRC screening dating back to the 80s. CRC screening in the US is not diffused in the form of organized programs but different test choices are equally recommended [i.e., (1) annual high-sensitivity gFOBT or FIT, following the manufacturer’s recommendations for specimen collection; (2) FS every 5 years; (3) TC every 10 years; (4) double-contrast barium enema every 5 years; or (5) CT colonography (CTC) every 5 years] (96). However, TC has progressively become the most widely used test in the US and is increasingly considered the gold standard test for CRC screening because of the claimed effectiveness in detecting cancer and advanced pre-malignant lesions in comparison with stool-based tests that primarily detect cancer at early stages (97–99). The preference of TC as a CRC screening test in the US is largely attributable to the classification of stool-based tests as aimed primarily at detecting cancer and as a test based on structural examination of large bowel as aimed at detecting both cancer and advanced pre-malignant lesions (96).

## Screening Strategies

Studies agree that at present there is no clear evidence of the superiority of one screening test and strategy over the others (28, 99).

High variability of screening interventions worldwide reflects this situation. Determinants of the adoption of screening strategies include: (i) test performance and, in particular, ability to detect pre-malignant lesions and decrease incidence of invasive cancer; (ii) acceptability of tests and screening participation; and (iii) resource needs associated with different strategies.

Fecal immunochemical test is increasingly considered a better test than gFOBT because of better accuracy, compliance, and cost-effectiveness (100). Moreover, FIT showed better sensitivity than gFOBT for advanced neoplasia and this feature should also result in a larger decrease of CRC incidence (18, 19, 101).

Among tests based on large bowel structural examination, TC is the preferred test, despite high costs and invasiveness. In particular, in the US, TC has progressively gained the widest diffusion over the other available tests, including FS (97). Endoscopy tests showed better sensitivity than FIT for the diagnosis of pre-malignant polyps and, particularly, advanced neoplasia. Thus, endoscopy tests are likely to confer better individual protection and have a stronger influence on cancer incidence if compared to FIT (17, 102). However, FIT is a better accepted screening test than FS and TC. Thus higher participation in screening using FIT may, to some extent, balance lower sensitivity than endoscopy tests (103–105).

Recent studies comparing FIT to endoscopy tests in a single screening round confirmed that FIT is associated with the highest participation (23, 101). Higher participation rates achieved by FIT reduce, but do not eliminate the gap in detection rate of cancer and AA compared to endoscopy tests. However, screening strategies using FIT have typically shorter intervals between subsequent test repetition that should further reduce or even reverse the difference in detection rates with respect to FS and TC (101). Results of studies comparing endoscopy tests with repeated rounds using FIT will depend on both test sensitivity and compliance with subsequent invitation in the FIT group (106, 107).

The diagnosis of pre-malignant lesions and consequent decrease of incident cancers is a much desirable feature of CRC screening. The concept of over-diagnosis apparently does not apply to CRC screening since a steeply increasing incidence does not follow screening introduction and, on the contrary, a decreasing incidence trend is reported in the US, after decades of CRC screening progressive diffusion (97). However, the concept of over-diagnosis in CRC should also be tested against incidence of pre-malignant lesions. In particular, the rate of colectomies should be monitored and compared among different screening tests and to non-screened groups or populations.

In conclusion, FIT testing is increasingly the most used CRC screening strategy. In a few countries, including US and Germany, TC is recommended and is the most used test. Cost and availability of endoscopy resources may be the limiting factors in the adoption of TC screening strategy.

## Improving Outcomes in CRC Screening

Improvement of CRC screening is a much desirable aim that is actively pursued through research to improve performance of tests and strategies to increase screening participation.

Despite its central role in CRC control and the availability of a range of effective tests, CRC screening is typically under-used. In the US participation in CRC screening has been increasing since its introduction in the 80s but was still below 70% in 2010 (97). In European countries, screening started much more recently than in the US and participation rates are generally lower than in US (13).

Strategies to improve participation in screening interventions were reviewed by Jepson et al. (108) and, more recently, by Camilloni et al. (109). The implementation of organized interventions may improve participation and reduce inequalities in screening uptake if compared to opportunistic screening (11). This strategy has been adopted in many European countries and is actively considered in Germany and US (13, 110, 111).

Physician recommendation influences participation (112). Sequential offering of available screening tests may contribute to maximize participation (113, 114). Research on screening tests with improved features including better acceptability is ongoing and serum markers represent a possible example of acceptable screening tests (115).

Better diagnostic performance is another way to improve CRC screening outcomes. Performance could be improved through: (i) strategies based on the combination of existing tests or (ii) improvement of screening tests that can be achieved with: (1) improvement of existing tests, (2) introduction of new diagnostic tests. CRC screening strategies currently in use consist either in opportunistic screening allowing individual selection of a test among many recommended alternatives or in organized screening based on the administration of a single type of test. The combination of tests with different features may be investigated to improve performance. Combination of FIT and FS has been discussed above. A simulation model showed that FIT at younger age combined with colonoscopy at older ages may represent a cost-effective alternative to single test strategies (116).

Research is continuously being done to improve existing tests or develop new ones. FIT can be considered an improvement of the older stool-based test gFOBT (100). As an example of innovation of existing tests, full-spectrum TC has recently been shown to have higher sensitivity for colorectal adenomas than traditional TC (117). Many new tests are currently in development. We briefly review tests that have been used or are ready for field use.

## Fecal DNA Testing

Fecal sample testing using molecular diagnostic tests is emerging as a potentially important new approach. There is a strong biologic rationale to pursue this technology, given that adenoma and cancer cells that contain altered DNA are continuously shed into the large bowel lumen. DNA is stable in the feces and it can therefore be extracted for analysis. Due to the heterogeneity of cancer, no single molecular marker has shown an optimal sensitivity, while panels of different markers in early studies have allowed a higher detection rate for both CCR and AA.

However, observations on larger size population studies appeared less encouraging, showing only fair sensitivity for the detection of CRC and low sensitivity for the detection of AA (118, 119). Significant technical improvements have been carried out in recent years, which have raised sensitivity of these tests for the detection of colonic lesions.

A very recent study by Imperiale (120) compared a multi-target stool DNA test with a commercial FIT among a large series of subjects at average-risk for CRC. The sensitivity of the DNA test for the detection of both CRC (92.3%) and advanced precancerous lesions (42.4%) was very impressive, being superior to that of FIT by a difference of about 20% points, even if FIT was more specific for CRC and advanced precancerous lesions (120). A higher rate of non-adequate samples respect to FIT (6.3 versus 0.3%) was also registered. DNA fecal testing is an addition to the stool-based tests for CRC screening but further studies are needed to understand whether stool DNA testing has any role in CRC organized screening, taking into account other key factors yet to be assessed, such as the screening interval, adherence, and costs.

Clinical proteomics is an emerging issue in cancer research. Potentially, blood proteomics tests in the near future will be able to detect patterns of proteins associated with cancer or low molecular-weight compounds related to an abnormal cell growth. In the words of Liotta and Petricoin (121) “the low-molecular-weight region of the blood proteome is a treasure trove of diagnostic information ready to be harvested by nanotechnology.” Unfortunately, no clinical applications are available at the moment and large multicenter studies in average-risk populations are needed in order to fully understand the true potential of this new biomolecular technology.

## CT Colonography

CT colonography, or virtual colonoscopy, is a poorly invasive radiological technique for imaging the large bowel. It provides two-dimensional and three-dimensional images. A bowel insufflation with carbon dioxide is needed. If polyps or CRC are detected on CTC, patients are referred to TC. CTC is well tolerated by patients (122, 123) and it can be performed even with limited bowel preparation. Several studies have demonstrated that CTC has a high-sensitivity for the detection of colonic lesions that was equal to 83–93% for polyps larger than 10 mm and to 60–86% for intermediate polyps with a 6–9 mm in size (124–129). Specificity of CTC resulted also very high for lesions >9 mm (95–97%) (125, 126, 128).

The risk of complications is extremely low, with no perforations or other serious complications in a large CTC screening cohort (130).

CT colonography reading may be time-consuming for the radiologist and this aspect is particularly interesting in a screening setting. Systems for helping radiologists in detecting colonic lesions at CTC have been developed [Computer aided diagnosis (CAD)]. Few data (131) suggest that this strategy has a sensitivity for polyps similar to that with unassisted reading and allows for a reduction in the reading time (132).

For these features, CTC could be a good alternative as primary test in a screening setting.

A RCT conducted in the Netherlands (133) compared participation in a CRC screening setting with CTC or with TC, showing that adherence of invited subjects was significantly better with CTC than with TC. On the other hand, TC identified significantly more advanced neoplasia per 100 participants than did CTC, even if the diagnostic yield for advanced neoplasia per 100 invitees was similar for both strategies.

In the perspective of including CTC as a screening test, some considerations about potential disadvantages are needed:

- CTC-based screening may produce a high referral rate to colonoscopy, with an increase of costs.
- Detection of extracolonic lesions: CTC displays the abdominal organs, thus the prevalence of extracolonic diseases that require further investigation may be substantial (6% in asymptomatic average-risk populations). Risks and costs associated with false-positive results and unnecessary diagnosis should be considered (134).
- Exposure of individuals to ionizing radiation; the probability of radiation-induced malignancy is very low, especially with low dose protocols.

Two randomized clinical trials are underway in Italy comparing CTC as primary test versus biennial FIT and versus TC (135) or versus FS (136). Both these trials will provide reliable information concerning participation/acceptability, diagnostic yield, and costs of screening with CTC in comparison with FIT or FS. The trials will also evaluate the role of CAD in a screening setting.

Pending the results of these studies, we currently have no available data about the effectiveness of CTC as primary test in CRC screening.

However, organized screening programs have already introduced CTC as a current complementary assessment for FIT positive patients with incomplete colonoscopy or with contraindications to colonoscopy (137).

The potential role of CTC as a first-line CRC screening strategy is very attractive. In this setting, CTC may offer clear advantages, such as accuracy, safety, and subject acceptance. Future research will tell us whether this strategy might be a good option in terms of participation, costs, and benefit/risks ratio for the CRC screening programs.

## Colon Capsule Endoscopy

Colon capsule endoscopy, also called video or wireless capsule endoscopy, is a relatively new technique to visualize the colon, developed by Given Imaging Ltd. for small bowel imaging. In 2006, a first generation of capsule, dedicated to colon investigation [PillCam Colon capsule endoscopy (CCE)] was developed. Images are transmitted to a computer workstation for the visualization. In case of abnormalities detected by CCE, a colonoscopy is needed to allow removal of polyps and subsequent pathologic diagnosis. Patients have to undergo bowel cleansing before the CCE. Bowel preparation is specifically designed not only to clean the colon, but also to allow colonic distension and propel the capsule through the colon. Even small amounts of residual stool may influence visualization of the colonic mucosa (138).

One study evaluated the diagnostic accuracy of CCE in a prospective setting with high-risk patients (139). Sensitivity and specificity for detecting polyps ≥6 mm was 64 and 84%, respectively, whereas sensitivity and specificity for advanced adenoma detection was 73 and 79%, respectively. Recently, a second-generation colon capsule (CCE-2) has been made available. The new CCE measures 11.6 mm × 31.5 mm, with a widened angle of view, thus allowing for nearly 360° coverage of the colon. The device can adapt the image acquisition rate depending on the speed of progression of the capsule along the colon. To further save battery energy, CCE-2 can “choose” to work at a low rate of 14 images per minute until small bowel images are detected.

CCE-2 is provided with a new data recorder (DR3) endowed with an artificial intelligence software. DR3 can communicate with the capsule that listens to the “thinking” Data Recorder 3 and carries out the orders received by it. Moreover, the DR3 guides the medical staff and the patient through the procedure, buzzing, and displaying instructions on its liquid crystal screen.

Two studies conducted in Israel (140) and in Italy (141) have evaluated CCE-2 diagnostic accuracy for polyps. In the first one, CCE-2 was prospectively compared with conventional colonoscopy as the gold standard in a cohort of 98 patients with known or suspected colonic disease. Per-patient CCE-2 sensitivity for polyps at least 6 mm in size was 89%, and at least 10 mm in size was 88%, with specificities of 76 and 89%, respectively. In the European trial, 109 patients were considered for analysis. Per-patient CCE-2 sensitivity for polyps at least 6 mm in size was 84% (95% CI 74–95%), and at least 10 mm in size was 88% (95% CI 76–99%) with a specificity of 64 and 95%, respectively. Data regarding diagnostic accuracy of the CCE-2 are encouraging, but evidence concerning the diagnostic performance of this new technology is in any case limited and based only on a few studies with a small number of subjects recruited. Moreover, studies in an average-risk screening population are still lacking. For this reason, a multicenter prospective study is underway in Italy with the aim of assessing the accuracy of PillCam colon 2 in a screening setting.

Colonic preparation for a colon capsule represents another challenge. Recent studies evaluated a new protocol with a split-dose PEG and a low dose of NaP, reporting good results (142).

In conclusion, the possible role of CCE as the primary test in CRC screening represents a fascinating perspective, but further studies are needed to understand the real impact of this new technique in the non-invasive diagnosis of CCR and its precursors.

## Conflict of Interest Statement

The authors declare that the research was conducted in the absence of any commercial or financial relationships that could be construed as a potential conflict of interest.
